# TMEM17 depresses invasion and metastasis in lung cancer cells via ERK signaling pathway

**DOI:** 10.18632/oncotarget.19977

**Published:** 2017-08-07

**Authors:** Xiupeng Zhang, Yong Zhang, Yuan Miao, Haijing Zhou, Guiyang Jiang, Enhua Wang

**Affiliations:** ^1^ Department of Pathology, College of Basic Medical Sciences and The First Affiliated Hospital, China Medical University, Shenyang, China; ^2^ Department of Pathology, Cancer Hospital of China Medical University, Shenyang, China

**Keywords:** TMEM17, lung cancer, ERK signaling, snail, occludin and Zo-1

## Abstract

Transmembrane protein 17(TMEM17) is a newly identified protein, its expression pattern and clinicopathological relevance is still unclear. In this study, western blot assay was performed in 20 paired lung cancer samples and found that TMEM17 protein levels were lower in lung cancer tissues than that in the corresponding normal lung tissues (p=0.010). Immunohistochemistry staining in 143 cases lung cancer specimens also showed that TMEM17 expression in lung cancer tissues were significantly lower than adjacent normal lung tissues (35.7% *vs* 63.2%, p<0.001). And negative TMEM17 expression was significantly associated with poor histological differentiation (p=0.027), advanced TNM stages (p=0.006), positive lymph node metastasis (p=0.002) and poor prognosis (p=0.002). After overexpressing TMEM17, levels of p-ERK and its downstream molecules, p-P90RSK and Snail, were down-regulated, while levels of Occludin and Zo-1 were up-regulated, which result in the inhibition of invasion and migration ability of lung cancer cells. The effects were reversed by the incorporation of specific ERK inhibitor PD98059. In conclusion, loss of TMEM17 correlates with the development of non-small cell lung cancer (NSCLC) and predicts adverse clinical outcome of NSCLC patients. The effect of TMEM17 on inhibiting invasion and migration may attribute to restoring Occludin and Zo-1 expression through inactivating ERK-P90RSK-Snail pathway.

## INTRODUCTION

Lung cancer is the most frequently diagnosed cancer and the leading cause of cancer death worldwide [1–3]. Tumor invasion and metastasis greatly limit treatment options and account for 90% of cancer-related death [4, 5]. Thus, it is important to search for new therapeutic targets involved in the regulation of lung cancer cell invasion.

Transmembrane protein 17 (TMEM17) is a newly identified 198 amino acid protein encoded by a gene mapping to human chromosome 2. TMEM17 is a cilium-associated protein, whose mutation display ciliogenesis defects [6]. Previous studies had confirmed that the primary cilium played a decisive role in the migration, the determination of cell polarity and cancer progression [7–9]. Therefore, we speculated that TMEM17 may be involved in the process. By using free online survival analysis software (KM Plotter Online Tool; http://www.kmplot.com), we found lower expression of TMEM17 significantly correlated with NSCLC patients poor survival (p=0.014, data not shown). However, to date, little is known on its expression pattern and molecular mechanism, especially in human lung cancer.

In this study, we explored the protein level and subcellular distribution of TMEM17 in both lung cancer tissues and cell lines, as well as their clinicopathological relevances. We also investigated the effects of TMEM17 on the invasiveness of NSCLC cell lines after TMEM17 overexpression or depletion. We found that TMEM17 depressed invasion of NSCLC cells through inhibiting the activation of ERK-p90RSK-Snail signaling pathway.

## RESULTS

### The expression of TMEM17 in NSCLC specimens

**Figure 1:**
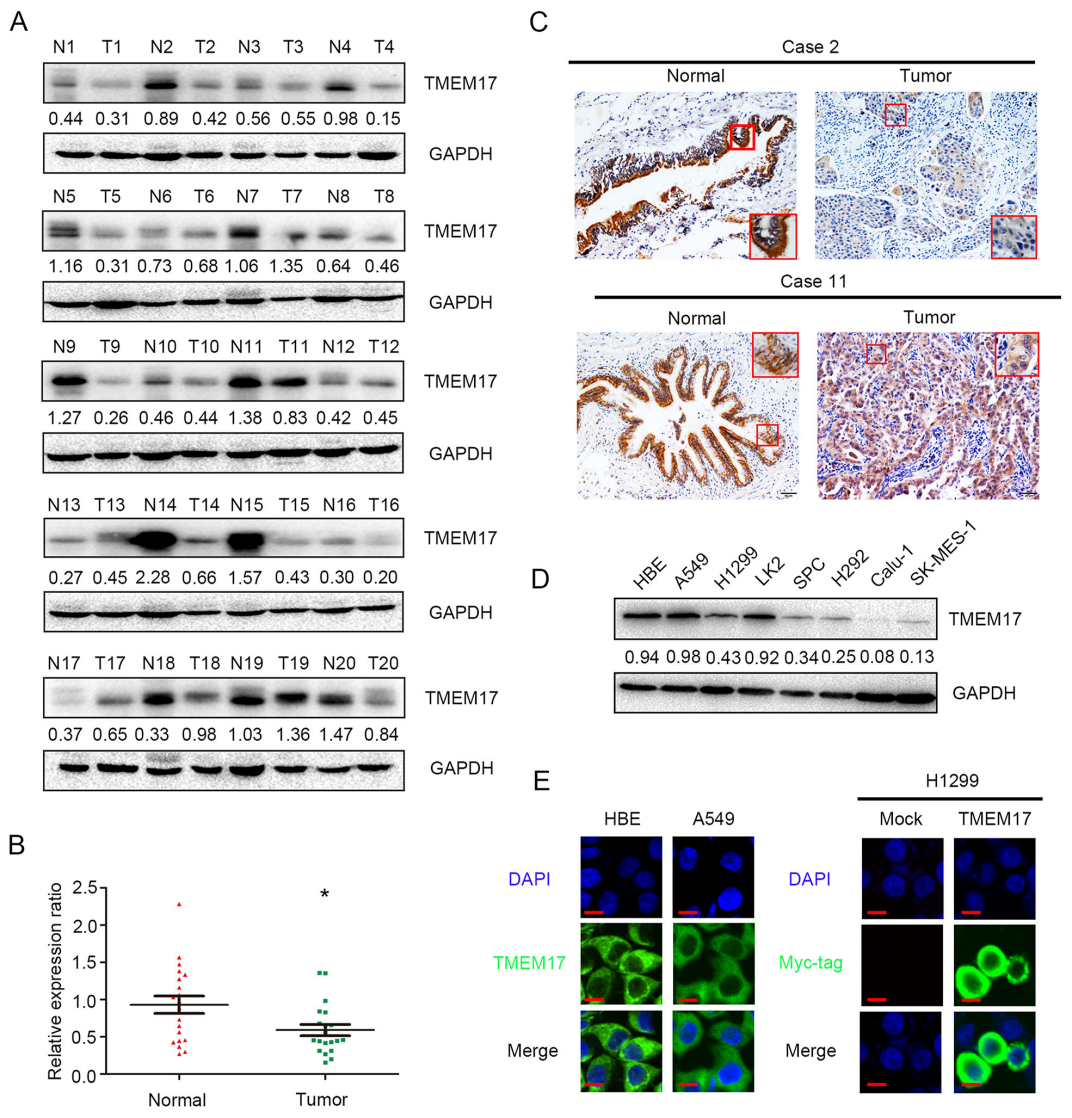
The expression of TMEM17 in NSCLC specimens **(A and B)**. TMEM17 was positively expressed in noncancerous tissues samples and only showed weak or negative expression in paired NSCLC samples. TMEM17 expression is significantly higher in normal lung tissue than that in NSCLC samples (P=0.010). (**C**, scale bar = 50 μm, insert scal bar=20μm) Representative images of IHC staining in cases 2, 10 and 11, TMEM17 showed cytosolic expression in all the cases. The expression of TMEM17 in HBE cells was higher than most of the NSCLC cell lines (except A549; **D**), and localized in the cytoplasm of HBE and A549 cells (**E**, left). Overexpressed TMEM17 is localized primarily in the cytoplasm in H1299 cells (detected by myc-tag, right). No positive signal is detected in non-transfected cells, 600× magnification

In the 20 cases of paired tissue samples, the results of western blotting showed that the normalized protein level of TMEM17 in noncancerous lung tissues (Mean ± SD = 0.9305 ± 0.1173) were significantly higher than in NSCLC specimens (Mean ± SD = 0.5902 ± 0.076, p=0.010, Figure 1A and 1B), and corresponding IHC staining results showed that TMEM17 was localized in the cytoplasm of all tested samples (Figure 1C).

In NSCLC cell lines and a normal bronchial epithelial cell line (HBE), WB and IF results showed that TMEM17 was highly expressed in HBE cells than the other NSCLC cell lines (Figure 1D), and localized in the cytoplasm (Figure 1E).

### Correlation between loss of TMEM17 expression and clinicopathological features of NSCLC

**Figure 2:**
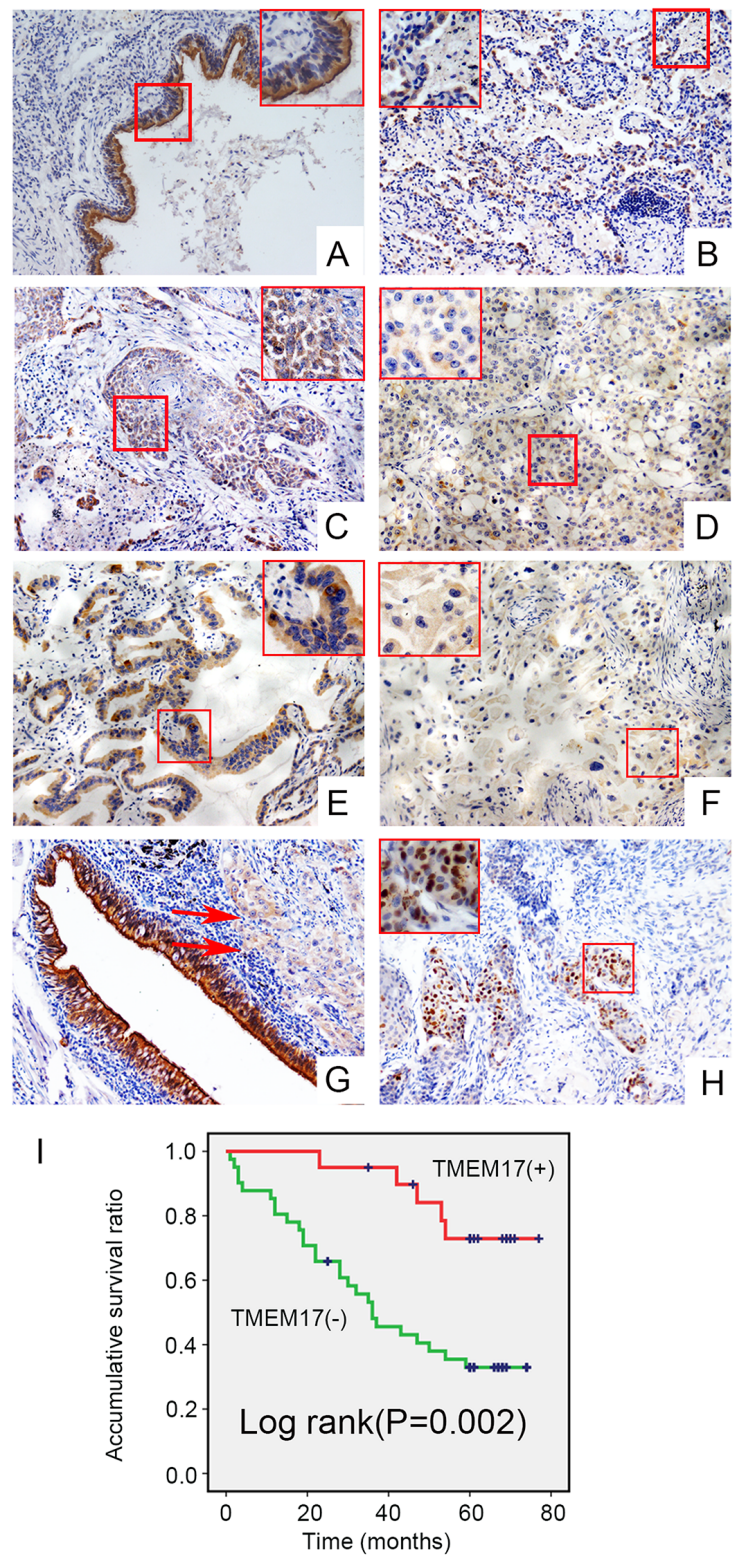
The expression and subcellular localization of TMEM17 in NSCLC tissues and cell lines Positive expression of TMEM17 in normal bronchial **(A)** and alveolar epithelial **(B)**, however, TMEM17 showed weak or negative expression in lung squamous cell carcinoma **(C-D)** and adenocarcinoma**(E-F)**. In the same case, TMEM17 was positive expressed in adjacent normal bronchial cells, while only showed weak expression in lung carcinoma (red arrow; **G**,). In rare cases, TMEM17 can be found expressing in the nucleus (**H**, scale bar = 50 μm, insert scal bar=20μm). Kaplan–Meier survival analysis revealed that the overall survival for patients with negative TMEM17 expression was significantly shorter than for those with positive TMEM17 expression **(I)**

The IHC staining results in 143 NSCLC specimens showed that TMEM17 expressed in the cytoplasm of normal bronchial and alveolar epithelial cells (Figure 2A and 2B) while presented only weak or negative expression in lung cancer tissues (Figure 2C-2F). The positive expression ratio of TMEM17 in normal lung tissues was obviously higher than that in NSCLC tissues (51/143, 35.7% vs 43/68, 63.2%, p<0.001, Figure 2E). Interestingly, TMEM17 can also be seen expressed in the nuclei in rare cases (5/143, 3.4%; Figure 2H). Statistical analysis indicated that loss of TMEM17 expression was significantly correlated with moderate or poor histological differentiation (p=0.027, Figure 2C and 2D, Figure 2E and 2F), advanced TNM staging (p=0.006) and positive lymph node metastasis (p=0.002) in NSCLC. In contrast, there was no significant association with sex, age, or NSCLC histological type (Table 1). Kaplan–Meier survival analysis revealed that the patients with negative TMEM17 expression presented shorter overall survival (40.541 ± 4.204 months) than those with positive TMEM17 expression (63.269±4.176 months, p=0.002; Figure 2I). Subsequent univariate and multivariate analyses found that negative TMEM17 expression had a tendency to be the independent prognostic factors in NSCLC (p=0.059, Table 2 ).

**Table 1:** Correlation of the expression of TMEM17 with clinicopathological features in 143 cases of NSCLC

Clinicopathological factors	N	Positive	Negative	χ2	*P*
Age (years)					
<61	53	19	34		
≥61	90	32	58	0.001	1.000
Gender					
Male	89	31	58		
Female	54	20	34	0.071	0.858
Histological type					
Squamous cell carcinoma	60	21	39		
Adenocarcinoma	81	30	51		
Large cell carcinoma	2	0	2	1.187	0.552
Differentiation					
Well	49	24	25		
Moderate+Poor	94	27	67	5.76	0.027
TNM classification					
I+II	70	33	37		
III	73	18	55	7.874	0.006
Lymph node metastasis					
Positive	77	18	59		
Negative	66	33	33	10.978	0.002

**Table 2:** Summary of Cox univariate and multivariate regression analysis of the association between clinicopathological features and overall survival in 61 cases of non-small cell lung cancer (NSCLC)

Clinicopathological feature	Hazard ratio (95% CI)	P
**Univariate analysis**		
Age older than 61	1.069 (0.515-2.219)	0.859
Gender: male	1.132 (0.565-2.268)	0.726
Histological type : Adenocarcinoma	1.244 (0.616-2.511)	0.542
Poor differentiation	1.799 (0.546-5.935)	0.355
High TNM classification	2.456 (1.208-4.997)	0.013
Positive lymph node metastasis	0.893 (0.441-1.811)	0.755
Negative TMEM17 expression	0.386 (0.173-0.861)	0.02
**Multivariate analysis**		
High TNM classification	2.090 (1.013-4.308)	0.046
Negative TMEM17 expression	0.455 (0.201-1.031)	0.059

### TMEM17 decreased invasion and migration of NSCLC cell lines

**Figure 3:**
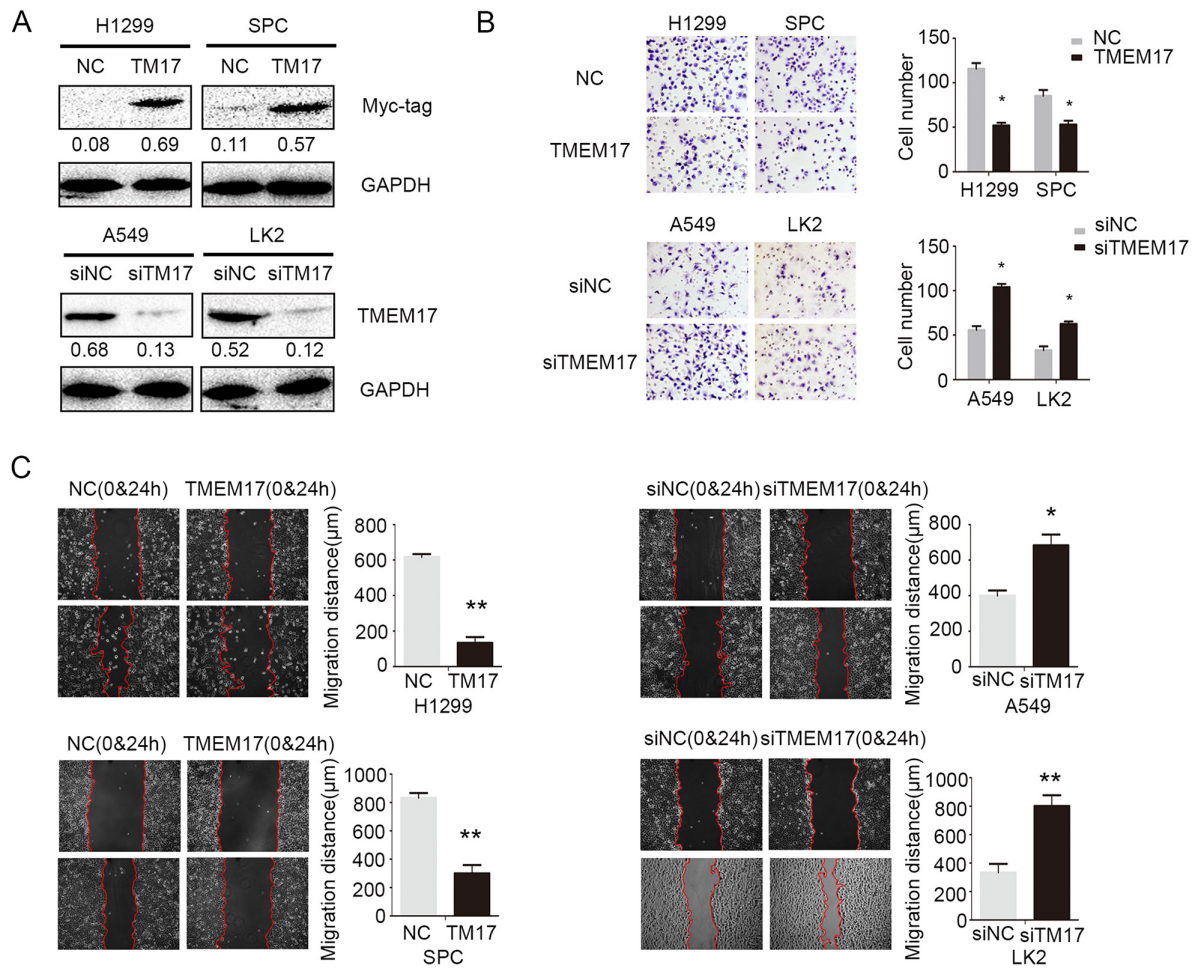
The effects on invasion and migration by overexpressing and depleting TMEM17 **(A)**. The transfection efficiency after overexpressing TMEM17 in H1299 and SPC cells and depleting TMEM17 by siRNA in A549 and LK2 cells were tested by western blotting. After overexpressing TMEM17 in H1299 and SPC, the invasion **(B)** and migration **(C)** was significantly depressed, after knockdown TMEM17 in A549 and LK2 cells, the invasion (B, scale bar = 50 μm) and migration (C) was obviously enhanced.T17 was short for TMEM17 (*P<0.05;**P<0.01).

We overexpressed TMEM17 and depleted TMEM17 by transfected TMEM17 cDNA into H1299 and SPC cells or tranfected TMEM17 siRNA into A549 and LK2 cells, respectively (Figure 3A). Matrigel invasion test results revealed that the invasiveness of H1299 (p=0.018) and SPC (p=0.037) was obviously decreased after overexpression of TMEM17 (Figure 3B), while the invasiveness of A549 (p=0.021) and LK2 (p=0.012) cells was significantly increased after depletion of TMEM17 (Figure 3B). Wound assay also indicated that the migration was depressed by overexpressing TMEM17 in H1299 (p=0.008) and SPC (p=0.004) cells and enhanced by depleting TMEM17 in A549 (p=0.042) and LK2 (p=0.001) cells (Figure 3C).

### TMEM17 decreased Snail and invasion of NSCLC cell through ERK signaling

**Figure 4:**
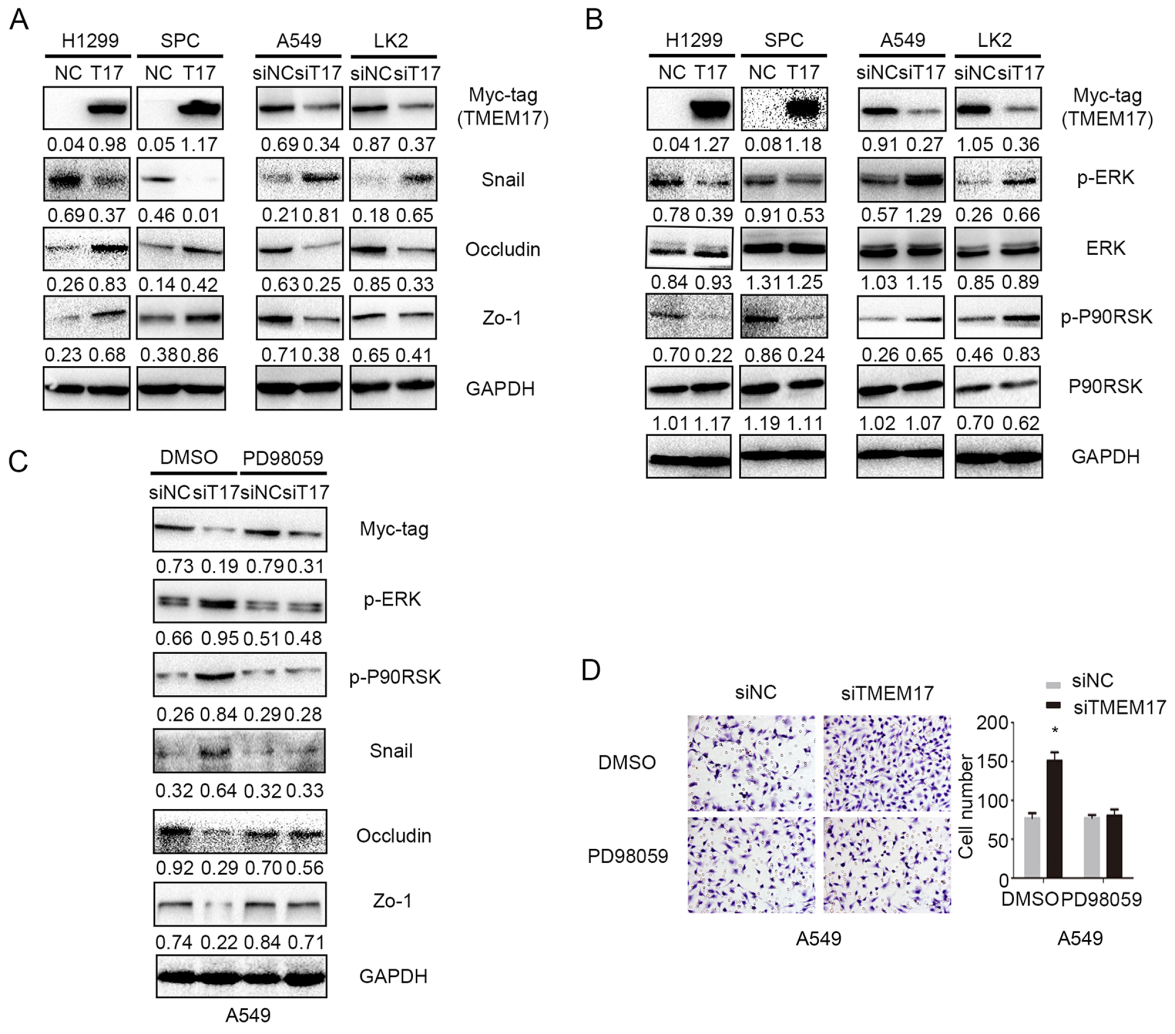
TMEM17 depressed NSCLC invasion and migration by ERK- p90RSK-Snail pathway **(A)** When overexpressing TMEM17 in H1299 and SPC cells, Snail was downregulated, Occludin and Zo-1 were upregulated. When depleting TMEM17 in A549 and LK2 cells, Snail expression was upregulated and Occludin and Zo-1 were downregulated. **(B)** TMEM17 overexpression downregulated the levels of p-ERK and its downstream factor p-P90RSK, and their expression were increased after knockdown TMEM17. **(C)** Western blotting examination of p-ERK, p-p90RSK, Snail, Occludin and Zo-1protein levels after transfecting TMEM17-siRNA with or without incorporation of PD98059. Representative images of cells on the bottom of Transwell membranes show the changes in invasive cell numbers (×200, scale bar= 50 μm) (*P< 0.05). T17 was short for TMEM17

As TMEM17 inhibited invasion and migration in lung cancer cells, we thereby examined common proteins involved in the process of controlling invasiveness of lung cancer after TMEM17 transfection and TMEM17 depletion. The results showed that Snail was downregulated, Occludin and Zo-1 were up-regulated when overexpressing TMEM17 in H1299 and SPC cells, and Snail was upregulated, Occludin and Zo-1 were downregulated when depleting in TMEM17 in A549 and LK2 cells. No visible changes were seen in the expression of other proteins such as E-cadherin, N-cadherin, Vimentin, Slug, Twsit, SIP1, MMP-2 and MMP-9 (Supplementary Figure 1A). We further detected the effect on the morphological changes after overexpression of TMEM17 in H1299 or inhibition of TMEM17 in A549 cells, the results revealed no visible alteration(Supplementary Figure 1B). After transfecting TMEM17 plasmid into H1299 and SPC cells or TMEM17 siRNA into A549 and LK2 cells, the protein and phosphorylated levels of ERK, p90RSK, p38, AKT, β-catenin and FAK were examined by western blotting. The levels of p-ERK and p-p90RSK, but not ERK and p90RSK, were obviously decreased after TMEM17 overexpression in H1299 and SPC cells and were elevated after TMEM17 knockdown in A549 and LK2 cells (Figure 4B). The expression of p-p38, p-AKT, active β-catenin and p-FAK showed no significant changes after TMEM17 overexpression in H1299 and SPC cells or TMEM17 depletion in A549 and LK2 cells (Supplementary Figure 1C). Subsequently, PD98059, an ERK inhibitor that specifically represses ERK phosphorylation, was added to the TMEM17-depleting cell lines. PD98059 reduced TMEM17 depletion induced p-ERK expression and in turn reduced p-p90RSK and Snail expression, while restoring the decreased expression of Occludin and Zo-1 in A549 cells (Figure 4C). PD98059 also diminished the increased invasive capacities caused by TMEM17 depletion (Figure 4D).

## DISCUSSION

Our study revealed that TMEM17 was highly expressed in the cytoplasm of normal lung tissue, while negatively expressed in lung cancer cells. Negative expression of TMEM17 significantly correlated with poor histological differentiation, high TNM stage, positive lymph node metastasis and poor prognosis. Although no evidences of the inhibitory role played by TMEM17 have been reported, there are multiple literatures about other family members of the transmembrane proteins [10]. In our study, despite the results of multivariate analysis only presented a tendency for TMEM17 to be an independent prognostic factor in NSCLC, we believed that it may attribute to relatively smaller sample size.

TMEM17 is a potential transmembrane protein containing four transmembrane domains (45-65aa, 78-98aa, 110-130aa, 142-162aa, respectively). The subcelluar distribution of TMEM17 was mainly in the cytoplasm. Interenstingly, we did not find any membranous TMEM17 in both NSCLC cell lines and specimens, but 5 of 143 cases presented nuclear staining in clinical tissue samples. To further detect the subcellular localization of TMEM17, we performed nuclear and cytoplasmic protein extraction and membrane and cytosol protein extraction in NSCLC cells. There results showed that endogenous and exogenous TMEM17 mainly localized in the cytoplasm (Supplementary Figure 2A and 2B), which was consisted with the immunofluorescence results. The reason why TMEM17 showed dominant cytosolic expression needed to be further clarified.

TMEM17 was found significantly inhibited migration and invasion in the present study. EMT (Epithelial-Mesenchymal Transition) associated proteins and MMPs were key regulators involved in NSCLC invasion and metastasis [11–15]. We discovered that TMEM17 inhibited Snail expression and restored Occludin and Zo-1 expression. However, the overexpression or inhibition of TMEM17 may not influence the morphological changes in H1299 or A549 cells. The results indicated that TMEM17 may only downregulate Snail and upregulate Occludin and Zo-1, but not the process of EMT. The detailed mechanism should be further investigated. In previous studies, the above proteins had been proven to be regulated by their upstream signaling pathways. Gupta GD et al. had reported that TMEM17 may directly interact with Dvl-1 (Dishevelled-1) and LRP6 (Low density lipoprotein related protein 6) which are crucial factor in canonical Wnt signaling pathway [16]. Our results revealed that active-β-catenin showed no significant alteration whenever we transfected or knockdown TMEM17 in NSCLC cells, which indicated TMEM17 impacted NSCLC cell invasion may be not induced by Wnt signaling pathway. Therefore, we screened for key factors of other common signaling pathways [17–25] and found that TMEM17 inhibited the phosphorylation of ERK signaling and its downstream P90RSK. Other signaling pathway displayed no obvious changes. Furthermore, inhibiting ERK activation through specific inhibitor diminished the effect of TMEM17 depletion on elevated expression of p-ERK, p-p90RSK and Snail, and also restored the reduced expression of Occludin and Zo-1 caused by TMEM17 depletion.

How TMEM17 modulate ERK signaling? Gupta et al had found that TMEM17 may interact with PAK4 [16], which may activate MEK, an enhancer of ERK signaling, thus promote ERK signaling [26]. We wonder the interaction between TMEM17 and PAK4 may interrupt ERK signaling. The hypothesis should be further tested in future.

In conclusion, TMEM17 was expressed in the cytoplasm of normal lung tissues or cells, and downregulated in lung cancer. Negative TMEM17 expression significantly correlated with poor histological differentiation, high TNM stage, positive lymph node metastasis and poor prognosis. TMEM17 inhibited Snail and enhanced Occludin and Zo-1 via inactivating ERK-P90RSK signaling, thus depressed invasion and migration of NSCLC cells.

## MATERIALS AND METHODS

### Patients and specimens

This study was approved by the local institutional review board of the China Medical University. Tissue samples were obtained from 143 patients (89 males and 54 females) who underwent complete surgical excision at the First Affiliated Hospital of China Medical University between 2010 and 2012 with a diagnosis of lung squamous cell carcinoma or lung adenocarcinoma. No neoadjuvant radiotherapy or chemotherapy was done before surgery, and all received standard chemotherapy after surgery. Of the 143 lung cancer cases, 61 contained complete follow-up data. The survival of each patient was defined as the time from the day of surgery to the end of follow-up or the day of death due to recurrence or metastasis. Histological diagnosis and grading were evaluated according to the 2015 World Health Organization (WHO) classification of tumors of lung [27]. All 143 specimens were for histological subtype, differentiation, and tumor stage. Tumor staging was performed according to the seventh edition of the International Union against Cancer (UICC) TNM Staging System for Lung Cancer [28]. The median age in 143 patients was 61 years old (range from 29 years old to 80 years old). Of the 143 patients, 53 patients were older than 61 years. The samples included 60 squamous cell lung carcinoma and 81 lung adenocarcinoma and 2 large cell lung carcinoma cases, respectively. A total of 49 tumors were well differentiated, while 94 were classified as moderately or poorly differentiated. Lymph node metastases were present in 77 of the 143 cases. The tumors included 70 stages I–II cases and 73 stage III cases. In addition, a total of 20 freshly isolated specimens, including both tumor and paired normal tissues, were stored at −80 °C immediately after resection for protein extraction.

### Western blotting (WB)

Total protein was extracted using a lysis buffer (Pierce, Rockford, IL, USA) and quantified with the Bradford method [29]. Fifty μg of the total protein samples were separated by 10% SDS-PAGE, and transferred onto polyvinylidene fluoride membranes (PVDF; Millipore, Billerica, MA, USA). Membranes were incubated overnight at 4°C with the following primary antibodies: TMEM17 and LaminB1 (1:100, sc-514433, Santa Cruz Biotechnology, Santa Cruz, CA, USA.), GAPDH (1:5000, Sigma, St. Louis, MO, USA), Snail, Slug, MMP2, MMP9, Myc-tag, p-ERK, ERK, p-P90RSK, P90RSK, p-AKT, AKT, p-FAK, FAK, Active-β-catenin and Vimentin (1:1000; Cell Signaling Technology, Danvers, MA, USA), E-cadherin, N-cadherin, β-catenin (1:1000; BD Transduction Laboratories, Lexington, KY, USA),ATPA1, Twist, Zo-1, and Occludin (1:500; Proteintech, Chicago, IL, USA),SIP1(Abcam, Cambridge, UK). α-tublin antibody (1:500) ,Membrane and Cytosol Protein Extraction Kit and Nuclear and Cytoplasmic Protein Extraction Kit were purchased from Beyotime (Jiangsu, China). Membranes were washed and subsequently incubated with peroxidase-conjugated anti-mouse or anti-rabbit IgG (Santa Cruz Biotechnology) at 37 °C for 2h. Bound proteins were visualized using electrochemiluminescence (Pierce, Rockford, IL, USA) and detected with a bio-imaging system (DNR Bio-Imaging Systems, Jerusalem, Israel).

### Immunohistochemistry (IHC)

Samples were fixed in 10% neutral formalin, embedded in paraffin, and sliced in 4-μm thick sections. Immunostaining was performed by the streptavidin-peroxidase method. The sections were incubated with a monoclonal mouse anti-TMEM17 antibody (1:100; Santa cruz) at 4°C overnight, followed by biotinylated goat anti-mouse IgG secondary antibody. After washing, the sections were incubated with horseradish peroxidase-conjugated streptavidin–biotin (Ultrasensitive; MaiXin, Fuzhou, China) and developed using 3,3-diaminobenzidine tetra-hydrochloride (MaiXin). Finally, samples were lightly counterstained with hematoxylin, dehydrated in alcohol, and mounted. Two investigators blinded to the clinical data semi-quantitatively scored the slides by evaluating the staining intensity and percentage of stained cells in representative areas. The staining intensity was scored as 0 (no signal), 1 (weak), 2 (moderate), or 3 (high). The percentage of cells stained was scored as 1 (1–25%), 2 (26–50%), 3 (51–75%), or 4 (76–100%). A final score of 0–12 was obtained by multiplying the intensity and percentage scores. Tumors were seen as positive TMEM17 expression with a score ≥4. Tumor samples with scores between 1 and 3 were categorized as showing weak expression, whereas those with scores of 0 were considered to have no expression; both weak expression and no expression were defined as negative TMEM17 expression.

### Cell culture

The HBE cell line was obtained from the American Type Culture Collection (ATCC; Manassas, VA, USA). The A549, H292, H1299, Calu-1, SK-MES-1 and SPC-A-1 (SPC) cell lines were obtained from the Shanghai Cell Bank (Shanghai, China). The LK2 cell line was a gift from Dr. Hiroshi Kijima (Department of Pathology and Bioscience, Hirosaki University Graduate School of Medicine, Japan). All cells were cultured in RPMI 1640 (Invitrogen, Carlsbad, CA, USA) supplemented with10% fetal bovine serum (Invitrogen), 100 IU/ml penicillin (Sigma), and 100 μg/ml streptomycin (Sigma), and passaged every other day using 0.25% trypsin (Invitrogen).

### Immunofluorescence (IF)

Cells were fixed with 4% paraformaldehyde, blocked with 1% BSA, and incubated with a monoclonal anti-TMEM17 antibody (1:50, Santa cruz) overnight at 4°C, followed by a tetramethylrhodamine isothiocyanate (TRITC)-conjugated secondary antibody added at room temperature for 1h. Cells were counterstained with 4′,6-diamidino-2-phenylindole (DAPI). Epifluorescence microscopy was performed using an inverted Nikon TE300 microscope (Melville, NY, USA); confocal microscopy was carried out on a Radiance 2000 laser scanning confocal microscope (Carl Zeiss, Thornwood, NY, USA).

### Plasmid transfection and small interfering RNA treatment

Plasmids pCMV6-ddk-myc and pCMV6-ddk-myc-TMEM17 were purchased from Origene (Rockville, MD, USA). TMEM17-siRNA (sc-94962) and NC-siRNA (sc-37007) was purchased from Santa Cruz Biotechnology. Transfection was carried out using the Lipofectamine 3000 reagent (Invitrogen) according to the manufacturer’s instructions.

### Matrigel invasion

Cell invasion assay was performed using a 24-well transwell chamber with 8 μm pores (Costar, Cambridge, MA, USA). The inserts were coated with 20μl Matrigel (1:3 dilution; BD Bioscience, San Jose, CA, USA). Forty-eight hours after transfection, cells were trypsinized, and 3×10^5^ cells in 100 μl of serum-free medium were transferred to the upper Matrigel chamber for 18h. Media supplemented with 10% FBS were added to the lower chamber as a chemoattractant. After incubation, cells that passed through the filter were fixed with 4% paraformaldehyde and stained with hematoxylin. The invasive cells were microscopically counted in ten randomly selected high-power fields.

### Wound healing assay

In cultures with cell density below 90%, 48 hours after transfection, wounds were created in confluent areas using a 200-μl pipette tip. Wound healing within the scrape line was observed at both 0h and 24h, and representative scrape lines for each cell line were photographed. Duplicate wells were examined for each condition, and each experiment was repeated 3 times. The optical wound distances were measured using Image J software (National Institute of Health, Bethesda, MD, USA).

### Statistical analysis

SPSS version 22.0 for windows (SPSS, Chicago, IL, USA) was used for all analyses. The Pearson’s Chi-square test was used to assess possible correlations between TMEM17 and clinicopathological factors. Kaplan–Meier survival analyses were carried out in 61 cases specimens and compared using the log-rank test. The Cox regression model was used to test the prognostic value. All of the clinicopathologic parameters were included in the Cox regression model and tested by univariate and multivariate analysis using enter method. Mann-Whitney U test was used for the image analysis of western blot results and the invasive assay results. *P*< 0.05 was considered to indicate statistically significant differences.

## SUPPLEMENTARY MATERIALS FIGURES


