# Supplementary material for: TMEM17 depresses invasion and metastasis in lung cancer cells via ERK signaling pathway
Source: Oncotarget. 2017 Aug 7;8(41):70685–94. doi: 10.18632/oncotarget.19977 (PMC5642586; doi:10.18632/oncotarget.19977)
Supplement: Supplementary file 1 [file oncotarget-08-70685-s001.pdf]

TMEM17 depresses invasion and metastasis in lung cancer cells via ERK signaling pathway

SUPPLEMENTARY MATERIALS

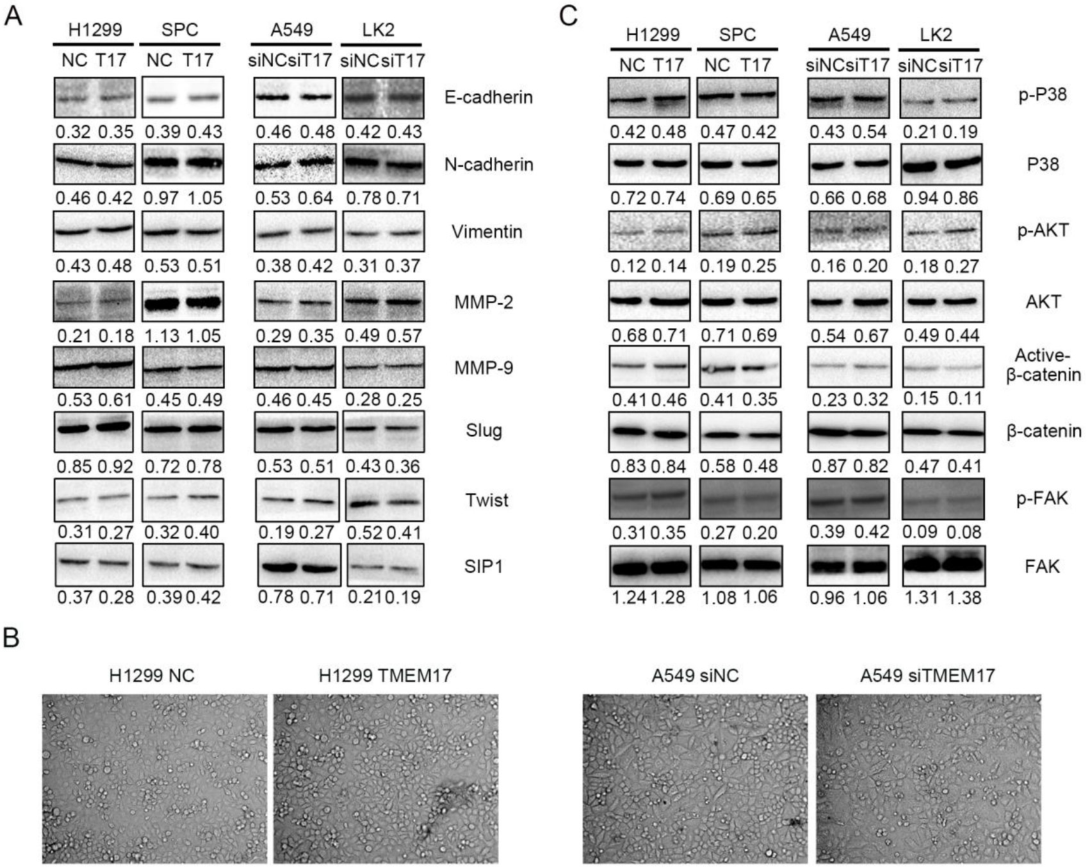

Supplementary Figure 1: The change of the expression of key proterins after modulating TMEM17. **(A)** E-cadherin, N-cadherin, Vimentin, MMP-2 and MMP-9 displayed no obvious changes after regulating the expression of TMEM17. **(C)**. The cell morphology also revealed no remarkable changes followed by overxpression or depletion of TMEM17(C) Other signaling pathway key proteins such as p-P38, p-AKT, Active-β-catenin, and p-FAK showed no obvious alterations after overexpressing or depleting TMEM17. T17 was short for TMEM17

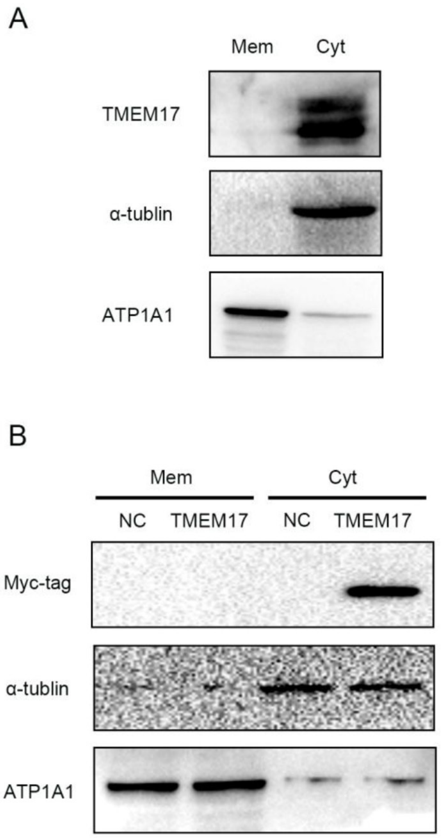

Supplementary Figure 2: The subcellular localization of TMEM17. We performed membrane and cytosol protein extraction (A and B, left) as well as cytosolic and nuclear protein extraction (A and B, right) in A549 **(A)** and H1299 **(B)** cells followed by TMEM17 depletion. The results suggested that both endogenous and exogenous TMEM17 mainly localized in the cytoplasm
